# Assessment of Hounsfield Units and Factors Associated with Fragmentation of Renal Stones by Extracorporeal Shock Wave Lithotripsy: A Computerized Tomography Study

**DOI:** 10.3390/tomography10010008

**Published:** 2024-01-11

**Authors:** Abdallah Saud Alharbi, Moawia Gameraddin, Awadia Gareeballah, Zahra Jibril Shrwani, Moa’ath Abdullah Sindi, Hassan Ibrahim Alsaedi, Abdulaziz A. Qurashi, Khalid M. Aloufi, Abdullah Fahad A. Alshamrani, Amel F. Alzain

**Affiliations:** 1 Medical Imaging Department, King Fahad Hospital, Al-Madinah 42210, Saudi Arabiazshrwani@moh.gov.sa (Z.J.S.);; 2 Department of Diagnostic Radiology Technology, College of Applied Medical Sciences, Taibah University, Al-Madinah 41477, Saudi Arabia; agsali@taibahu.edu.sa (A.G.);; 3 Department of Diagnostic Radiology, Faculty of Radiological Sciences and Medical Imaging, Alzaiem Alazhari University, Khartoum 13311, Sudan; 4 Medical Imaging Department, King Abdulaziz Medical City, Jeddah 21423, Saudi Arabia

**Keywords:** fragmentation, CT number, renal stones, ESWL, serum calcium, uric acid

## Abstract

The success rate of extracorporeal shock wave lithotripsy (ESWL) is influenced by various factors, including stone density, and is determined through computed tomography scans in terms of Hounsfield units (HU). Materials and Methods: This retrospective single-center study was conducted in the King Fahad Hospital. Sixty-seven adult patients with renal and ureteric stones were selected randomly and enrolled in the study. Their ages ranged from 20 to 69 years. The patients were examined with non-contrast enhancement (NCCT) to assess the HU of their stones and were consequently treated with ESWL. Results: Of the 67 patients, 37.3% had stones that were completely fragmented, while 62.7% had stones that were partially fragmented. The HU, location of the stone, multiplicity of the stone, and patient age were found to be significant factors contributing to stone fragility (*p*-values < 0.05). The HU data were found to have a positive significant linear correlation with serum calcium (r = 0.28, *p*-value = 0.036), while serum acid had a negative correlation (r = −0.55, *p*-value < 0.001). Thus, the probability of calcium-containing stone formation increases with increased HU. In contrast, uric acid stone formation likely develops with decreasing HU with serum uric acid. Renal stones in patients with diabetes mellitus and hypertension were not completely fragmented compared to those without clinical history. Conclusions: Mean HU, location of the stone, laterality, stone status, and the number of ESWL sessions are the most significant factors affecting stone fragility. CT attenuation values can predict the composition of stones from serum calcium and uric acid examinations. Hypertension and diabetes mellitus are risk factors for renal stone fragmentation.

## 1. Introduction

Extracorporeal shock wave lithotripsy (ESWL) is an effective procedure for treating renal and ureteral stones [1]. Most renal and upper ureteric stones respond well to extracorporeal shock wave lithotripsy (ESWL), particularly those with a size range of 10–20 mm [2]. This therapeutic approach has a 60–90% success rate across several series [3,4,5]. However, several parameters, such as the size, location, composition, and existence of obstructions or infections, affect how well the ESWL treatment works [4,5]. Cysteine and calcium oxalate monohydrate stones are typically resistant to ESWL. Understanding stone composition is crucial for ESWL treatment, but doing so before treatment is complex.

The use of spiral/helical non-contrast computed tomography (CT) in individuals with urinary system stones (CT) has grown recently. Dual-energy computed tomography (DECT) technology is an exciting advancement over contrast-enhanced computed tomography (CECT). DECT, one of the most promising advances in radiography, is used to determine the components of various materials or to identify them [6]. It allows for a more accurate classification of renal stones regarding stone burden, composition, and fragility [6]. Previous studies have shown that DECT has clinical benefits in diagnosing renal stone types [7,8]. According to the current findings, DECT can identify pure uric acid, cystine, and calcium oxalate stone types with great sensitivity [9]. In the literature, scoring systems are applied to predict operation success with urinary system stones, to advise patients, and to standardize academic study reporting [10]. Some nephrolithometric scoring systems have been created in earlier investigations using preoperative clinical data and stone features. Initially, nomograms predicting stone operation outcomes were developed [11].

The density of the stone or structure of interest is connected to Hounsfield units (HU), a parameter produced from standard CT [12]. The CT number of urinary stones can predict the composition of the stones. CT is considered the gold standard for evaluating stones before surgery, and it affects surgical plan decisions [13]. The density of renal calculi is determined using HU, which also identifies high-density stones that should not be subjected to shockwave lithotripsy (SWL) [14]. The stone size, location, diversity, and HU values evaluated via non-contrast computed tomography (NCCT) are just a few of the variables reported to predict the success of ESWL [2,15]. Therefore, it is crucial to identify the patients who would benefit from ESWL before starting treatment.

Most urinary stones are well known to be composed of a complex mixture of numerous components, primarily calcium, oxalate, phosphate, and uric acid, in varying amounts, which can affect the composition and density of stones in different places. Most of the research that compared stone fragility to stone density assumed that the stones were formed from a single material and had consistent density throughout. As a result, the maximum or mean attenuation values in a few places of the research zone were determined and compared to the ESWL success rate. This will likely change the results of the NCCT comparison of stone fragmentability vs. stone density. Thus, different studies showed that the attenuation values of stones measured in HU were an effective way to determine the fragility of stones by ESWL. However, because most urinary stones contain a mixture of one or more minerals, the attenuation values determined by NCCT may vary greatly in different locations of the stone. The consideration of a single HU value or the mean value taken from a few locations of the stone is dubious. Furthermore, currently, more effective approaches such as helical NCCT can diagnose urinary stones with sensitivity and specificity, with an accuracy of more than 95% [16].

The current literature reveals that different predictors connected to the stone and the patient may influence stone fragmentation and clearance rates [17,18]. Clinical characteristics such as BMI, as well as computed tomography parameters such as stone position, skin-to-stone distance, diameter or stone volume, and HU are used as predictors [18,19].

This study aims to determine the outcome of ESWL of urolithiasis using CT attenuation (HU) for the prediction of stone fragmentation, and to identify the associated risk factors such as stone size, location, and laboratory investigations (serum calcium and serum uric acid).

## 2. Materials and Methods

This study conducted retrospective type research on a database of patients treated for renal and ureteral stones from March to December 2021 and evaluated 67 patients (56 males and 11 females) who were examined with no-contrast CT (NCCT) before ESWL at the King Fahd Hospital. More than one session of ESWL was conducted. The ESWL outcomes were determined in terms of the complete or partial fragmentation of renal stones regardless of stone-free status. The inclusion criteria were adults ≥ 20 years old with multiple or solitary renal or ureteral stones. The exclusion criteria were pregnancy, febrile urinary tract infections (UTIs), using anticoagulant treatment, and those who were less than 20 years old. Patients with thyroid diseases were excluded from the study.

### 2.1. CT Renal Protocol

The NCCT examination protocol of the renal system was applied using General Electric GE Pride Speed (MDCT) to assess the renal stones and estimate HU values. The following patients’ characteristics were considered: patient age, sex, stone laterality, stone length, stone side, mean and peak HU values, and stone position. The CT renal protocol was performed following the standard CT renal protocol. Scout was first taken in AP to map the CT volume’s precise distance. From the level of the diaphragm down to just below the symphysis pubis, a single volume of CT series was collected. The patient was prone, with feet first. A voltage of 120 kV was used, with 50 mAs; the scan time took 10 to 12 s; and the delay with one breath hold was 4 s. The slice thickness was 2 mm, and the gantry was not tilted. The chosen kernel was standard for soft tissue. The CT images were delivered to a dedicated workstation, a photo archiving computer system (PACS). The images are observed using curved multiplanar and 3D volume rendering techniques with axial, coronal, sagittal, and oblique reconstruction. Techniques involving maximum intensity and medium volume were frequently employed. Two radiologists reviewed the images, discussed the findings with the urologist, and agreed on the results.

### 2.2. Statistical Analysis

The data were collected using a designed data collecting sheet and analyzed using IBM SPSS for Windows, version 23 (IBM Corp., Armonk, NY, USA). Due to missing data, several cases were omitted from the study. Most of the missing data were related to the patient’s laboratory investigations. The study variables were categorical and quantitative. Descriptive statistics such as percentages and frequency distribution were used to describe the qualitative variables. Linear regression and Pearson’s correlation statistical tests were used to find the relationship between CT number with serum calcium and uric acid. Binary logistic regression analyses were conducted to estimate the associated risk factors and to find factors related to the fragmentation of renal stones, such as HU, stone location, stone length, and the number of ESWL sessions. The results of the modeling process also incorporated all relevant components connected to the outcome variable, such as age, sex, and clinical history.

In contrast to the chosen referent, the crude odd ratio (COR) and adjusted odds ratios (AOR) and their respective 95% confidence intervals (CI) were provided to assess the contribution of each factor to the outcome of stone fragmentation.

## 3. Results

A total of 67 patients were retrospectively reviewed to assess the stone fragmentation. Patient characteristics are shown in Table 1. Of the participants, 68.7% had a history of diabetes mellitus, while 28.4% had primary hypertension (Table 1). The prevalence of renal stones was higher in males (56, 83.6%) than in females (11, 16.4%), and higher in the age groups of 41–50 years (38.8%) and 31–40 years (20.9%).

Table 2 summarizes the status of renal stones treated with ESWL with a degree of hydronephrosis. Most of the stones were single (67.2%), 44.8% were located in the calyces of the kidney, and 35.8% were in the upper ureter. Locations in the lower and mid-ureter were less frequent. Most treated stones were found in the left and right kidneys (40.3% and 37.3%, respectively).

**Figure 1:**
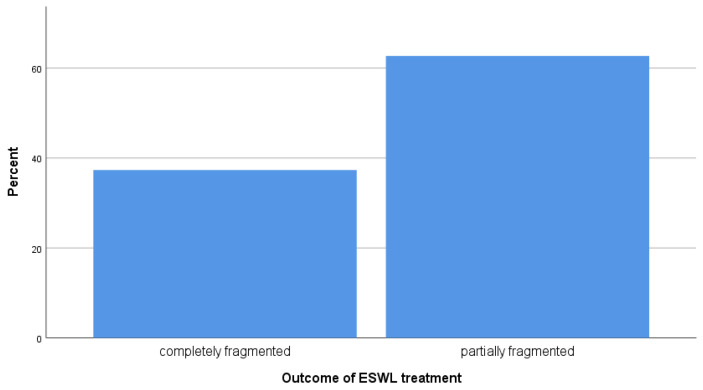
Outcomes of ESWL for renal stone fragmentation in the study population.

**Figure 2:**
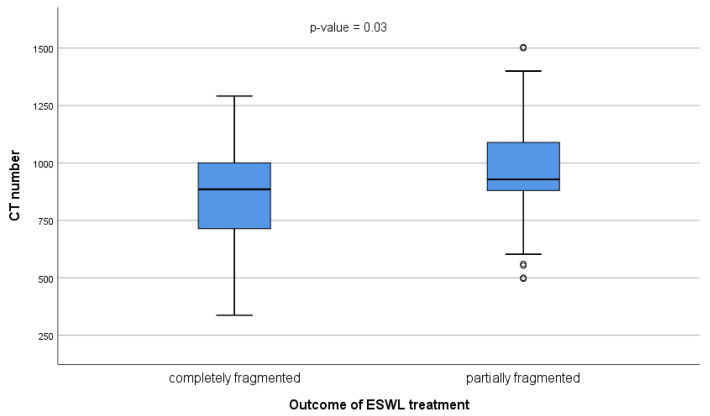
Relationships between CT attenuation values and ESWL outcomes for renal stone fragmentation.

The ESWL stone treatment outcomes were 37.3% completely fragmented and 62.7% partially fragmented (Figure 1). The mean CT attenuation number was significantly higher in partially fragmented than in completely fragmented stones (978.79 vs. 841.95, *p*-value = 0.03), as shown in Figure 2. These findings indicate that the CT attenuation numbers govern successful treatment via ESWL, since attenuation values below 841.95 HU may predict success, and values of ≥978.79 HU may lead to the failure of ESWL.

**Figure 3:**
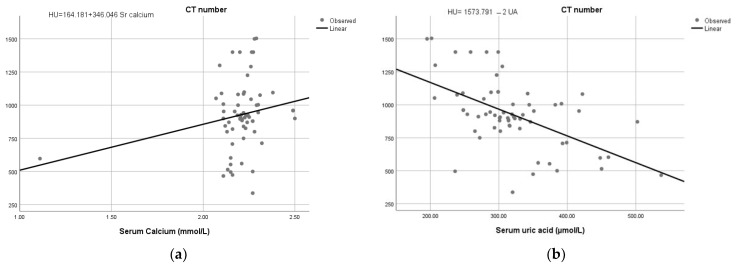
The regression estimation curve shows a positive relationship for CT number with serum calcium (**a**) and a negative linear relationship with serum uric acid (**b**).

There was a significant linear relationship for CT attenuation with serum calcium and uric acid values (*p*-values = 0.036 and <0.001, respectively) (Table 3). The correlation with serum calcium was positive (r = 0.28). At the same time, it was negative with serum uric acid (r = −0.51). Since there was a positive correlation between the variables mentioned above, we derived a regression equation for predicting the type of the stone with CT attenuation values and serum calcium and uric acid that had minimal error (Figure 3 and Table 3). The linear regression equation revealed that an increase in the CT number of a stone was associated with an increased likelihood of exhibiting calcium stones. In contrast, a decreased CT number was associated with a reduced probability of predicting uric acid stones.

The outcome of ESWL treatment (stone fragmentation) was distributed according to the stone location and kidney side, regardless of stone clearance status. Twenty-two stones in the calyces and renal pelvis were partially fragmented, and only eight were completely fragmented. Ten stones in the upper ureter were completely fragmented, and fourteen were partially fragmented (Table 4). It was shown that 19 cases of stones in the right kidney were partially fragmented, while 6 were completely fragmented. The left kidney showed 16 partially fragmented and 11 completely fragmented cases. The outcome in the right kidney is higher than in the left kidney.

The factors that predict stone fragmentation are summarized in Table 5, according to binary logistic regression. Aging is a significant factor for stone fragmentation (AOR = 1.29, 95% CI = 1.0–1.66). Males had 6.280 increased odds compared to females to respond to stone fragmentation (OR = 6.280, 95% CI = 0.202–195.027). Stones in the right kidney had 3.619 increased odds of being fragmented compared to stones located bilaterally (COR = 3.619, 95% CI = 0.921–14.214), while stones in the left kidney had 1.662 increased odds compared to those found bilaterally (COR –1.662, 95% CI = 0.466–5.932). The mean stone length is a significant factor for stone fragmentation (AOR = 1.037, 95% CI = 0.968–1.11). On the other hand, the site of the stone is also a significant factor contributing to fragmentation (AOR = 0.142, 95% CI = 0.032–0.626).

The HU is an essential factor associated with stone fragmentation. It had 1.003 increased odds compared to stones with lesser HU values (AOR = 1.003, 95% CI = 1.000–1.006). Regarding the number of ESWL treatment sessions, patients exposed to more than one session are more likely to respond to the fragmentation process than those with fewer sessions (AOR = 0.121, AOR = 0.023–0.646).

Considering the clinical history of the patients, the results revealed that diabetic patients had 1.406 increased odds compared to non-diabetic patients to respond to stone fragmentation (OR = 1.406, 95% CI = 0.489–4.043), while hypertensive patients had 1.8 increased odds compared to non-diabetic patients to respond to stone fragmentation (OR = 1.8, 95% CI = 0.713–3.208). The stones in people with diabetes are 1.406 times more partially fragmented than in non-diabetic patients, where stones are easily fragmented.

## 4. Discussion

CT is the most sensitive and reliable imaging technique for detecting urinary calculi. It can assess small radiolucent stones and other illnesses affecting the urinary system or other organs. Numerous studies have attempted to use factors like HU, skin-to-stone distance (SSD), and stone size to predict the composition and fragility of stones with CT [20,21]. The best parameter cutoff values that can forecast stone clearance are of clinical importance, particularly the cutoff values for the most potent predictive factors, since they may help select a treatment plan. We studied several factors that affected the fragility of renal stones. The laterality of the stone, status of the stone, stone location, and clinical history were the most significant factors predicting the fragmentation of renal stones. This study differs from others in that we included the clinical history, such as diabetes and hypertension, as risk factors for stone fragmentation, as well as other factors, such as stone location, stone length, stone status (single or multiple), HU, gender, and age.

This study showed that the stone’s laterality, status, and stone location were significant factors affecting the fragility of renal stones by ESWL. According to previous studies [22,23], the location of the stone is a reliable indicator of ESWL outcome. Our analysis examined the locations of renal and ureter stones on the calyces or renal pelvis, or the proximal, middle, and lower ureters. Many studies focused only on the location of the kidney. It was found that laterality is a significant factor in stone fragility. Thus, it was found that stones in the right or left kidney were more likely to respond to fragmentation than those located in both kidneys. To our knowledge, this finding has yet to be reported in a study.

This study found that the stone status, multiple or single, was a significant predictor for ESWL outcome. Our analysis found that numerous stones are more difficult to fragment than a single stone. In agreement with this finding, Ozgor et al. reported that after a single session of laser lithotripsy, patients with solitary kidney stones had a considerably higher stone-free status [24].

This study showed that the stone length is not a significant predicting factor for stone fragmentation, although the stone volume is considered an essential factor in the literature. In a study of individuals with solitary upper urinary tract calculi, Bandi et al. reported that stone volume was the best predictor of ESWL outcome. However, they also detected significant variations in axial diameters [25]. According to Nakasato et al., large stones frequently stay removed [26]. Large stones are ordinary, and have larger diameters than small ones; thus, they remain unchanged. Therefore, there are better ways to determine the ESWL outcome than classifying stones by length or size.

Regarding the CT number of the renal stone, this study found a significant association with the fragmentation process, either partially or entirely. Stones with higher CT numbers are partially fragmented compared to those with low CT numbers, which are completely fragmented. Consistently, our study found that complete fragmentation occurs in stones with HU of 841.95 and 978.79 for partial, fragmented stones. Several studies have reported the impact of HU on stone fragmentation. Wang et al. provided cutoff values of stone density > 900 HU [27]; Park et al. reported that an 863 HU threshold was the most significant predictor of ESWL outcome among the factors examined [28]; and Ouzaid et al. reported a 970 HU threshold [14]. Additionally, different studies reported that the attenuation values of stones measured in HU are an effective way to determine the fragility of stones by ESWL [14,15,16]. However, because most urinary stones contain a mixture of one or more minerals, the attenuation values determined by NCCT may vary greatly in different locations of the stone. The consideration of a single HU value or a mean value taken from locations of the stone is practicable and useful [16]. Therefore, CT attenuation values can help distinguish between stones that are expected to fragment quickly with ESWL and stones that would need more shock waves to disintegrate or not fragment with ESWL.

Regarding the prediction of stone composition, the present study revealed a significant linear relationship between CT number with serum calcium and uric acid examinations. A significant linear correlation existed between CT number, serum calcium, and uric acid. It is important to note that calcium nephrolithiasis can occur in people with hypercalcemia/hypercalciuria and those with normocalcemia/normocalciuria, even though calcium makes up the majority of crystalline components of kidney stones in 80% of instances [29]. This study found that serum calcium values in patients with renal stones increased the CT number, yielding a statistically positive linear correlation. This finding is consistent with Silva and Lima, who studied the “Correlation between HU values and stone composition in nephrolithiasis” and found that a higher HU value and greater age increased the likelihood of a stone being formed from calcium oxalate monohydrate [30]. In the literature, it was reported that hypercalcemia and hypercalciuria produce calcium nephrolithiasis [29,31]. It was effectively utilized to identify uric acid stones with comparable precision. Therefore, increasing CT attenuation values are associated with serum calcium, thus predicting calcium stones.

On the other hand, we found that serum uric acid decreases the CT attenuation values. In agreement with this finding, a study reported that uric acid stones exhibit no change in CT number [32]. Euler et al. consistently reported that CT attenuation of uric acid stones decreased significantly compared to non-uric acid stones (*p* < 0.001). They concluded that DECT accurately distinguished uric acid from non-uric acid stones, even with small stone sizes [33]. The findings supported that CT effectively discriminates uric acid urinary stones from non-uric acid stones [34,35]. Therefore, decreasing attenuation values predict uric acid stones as they have lower attenuation values compared to stones made of calcium salts. The relationship of serum calcium and uric acid with the HU of the stones needs a comprehensive investigation to predict the type of renal stones.

Epidemiologically, previous studies reported that diabetes and hypertension are risk factors for the formation of stones, although they did not affect the number of stones [36,37]. The present study found a relationship between stone fragility, diabetes, and hypertension. It was found that stones in diabetic and hypertensive patients are not easily fragmented compared to those of non-diabetic and non-hypertensive patients whose stones are entirely fragmented. No studies in the literature verified the impact of diabetes mellitus and hypertension on the fragmentation of renal stones. This finding needs comprehensive investigation. Although shock wave lithotripsy is less invasive, previous studies suggested that it may raise patients’ risk of hypertension and diabetes mellitus in the future [38,39].

This study had significant and minor limitations, as the sample size is not large enough, which may yield some statistical errors and bias. Secondly, no other technique was utilized to determine the composition of the stone such as chemical analysis, although the routine lab investigation is performed for the evaluation of extracted stones. So, the correlation of serum calcium with the HU might be affected in the statistical analysis. Further studies with larger samples are recommended to confirm these findings.

## 5. Conclusions

Many factors influence the outcome of ESWL for renal stone fragmentation. Mean HU, the location of the stone, laterality, the status of stone, and the number of ESWL sessions were the most significant factors affecting stone fragility. CT attenuation values can predict the composition of stones from serum calcium and uric acid examinations. Diabetes mellitus and hypertension are risk factors for renal stone fragmentation. To support future research and assist clinicians in making decisions, the prospective standardization of the HU measurement and patient history for shockwave lithotripsy outcomes should be considered.

## Figures and Tables

**Table 1:** Sociodemographic characteristics of the study sample.

Character	Frequency	Percent
Male	56	83.6
Female	11	16.4
Age groups (years)		
20–30	9	13.4
31–40	14	20.9
41–50	26	38.8
51–60	6	9.0
>60	12	17.9
Mean age = 45.57 ± 13.8		
Occupation		
employer	47	70.1
non-employer	20	29.9
Diabetes mellitus		
yes	21	31.3
no	46	68.7
Hypertension		
yes	19	28.4
no	48	71.6

**Table 2:** Status of renal stones treated using ESWL.

Status of Renal Stones	Frequency	Percent %
single	45	67.2
multiple	22	32.8
Site of stone in the kidney		
calyces of the kidney	30	44.8
upper ureter	24	35.8
lower ureter	7	10.4
mid-ureter	6	9.0
Hydronephrosis		
No hydronephrosis	16	23.9
mild	35	52.2
moderate	14	20.9
severe	2	3.0
Laterality		
right kidney	25	37.3
left kidney	27	40.3
bilateral	15	22.4

**Table 3:** Relationship of CT number with serum calcium and uric acid examinations using Pearson’s correlation and linear regression tests.

Characteristic	Statistical Correlation	Serum Calcium (mmol/L)	Serum Uric Acid (µmol/L)	Regression Equation
CT number	Pearson’s correlation	0.28 *	−0.55 **	HU=1477.647+38.742 Ca −1.99 uric acid
	Significant two-tailed	0.036	<0.001	

* Correlation is significant at the 0.05 level (two-tailed). ** Correlation is significant at the 0.01 level (two-tailed).

**Table 4:** ESWL treatment outcomes according to stone status.

Stone Location	Number of Stone	Completely Fragmented	Partially Fragmented
calyces and renal pelvis	30	8	22
upper ureter	24	10	14
mid-ureter	6	4	2
lower ureter	7	3	4
laterality			
right kidney	25	6	19
left kidney	27	11	16
bilateral	15	8	7
status of stone			
single	45	18	27
multiple	22	7	22

**Table 5:** Binary logistic regression analysis for factors that predict stone fragmentation by ESWL.

Variables	COR (95% CI)	AOR (95% CI)
Mean age (SD)	0.993 (0.958–1.029	* 1.29 (1.0–1.66)
Gender (n)		
Males	2.34 (0.631–8.656)	6.280 (0.202–195.027)
Females	Ref	Ref
Clinical history		
Diabetes mellitus	1.406 (0.489–4.043)	3.981 (0.343–46.240)
Hypertension	1.8 (0.713–3.208)	4.354 (0.436–1.488)
Laterality		
Right kidney	3.619 (0.921–14.214)	8.261 (0.396–172.531)
Left kidney	1.662 (0.466–5.932)	* 22.338 (1.003–497.552)
Bilateral	Ref	Ref
Site of the stone within the kidney	0.599 (0.349–1.029)	* 0.24 (0.079–0.731)
Mean of HU (SD)	* 1.002(1–1.005)	* 1.003 (1.000–1.006)
Status of stone		
Multiple	1.120 (0.803–1.562)	* 3.516 (0.398–31.036)
Single	Ref	-
Mean of stone length (SD)	1.035 (0.980–1.093)	1.078 (0.973–1.193)
Frequency of ESWL treatment sessions	0.592 (0.293–1.195)	* 0.121(0.023–0.646)

* Significance > 0.001.

## Data Availability

Data supporting the reported results are available upon request from the study’s corresponding author.
